# Lack of effect of glutamine administration to boost the innate immune system response in trauma patients in the intensive care unit

**DOI:** 10.1186/cc9388

**Published:** 2010-12-24

**Authors:** Jon Pérez-Bárcena, Catalina Crespí, Verónica Regueiro, Pedro Marsé, Joan M Raurich, Jordi Ibáñez, Abelardo García de Lorenzo-Mateos, José A Bengoechea

**Affiliations:** 1 Intensive Care Medicine Department, Son Dureta University Hospital, Andrea Doria 55, 07014, Palma de Mallorca, Spain; 2 Cátedra de Medicina Crítica, Departamento de Cirugía, Universidad Autónoma de Madrid, Arzobispo Morcillo 2, 28029, Madrid, Spain; 3 Research Unit, Son Dureta University Hospital, Palma de Mallorca, Andrea Doria 55, 07014, Palma de Mallorca, Spain; 4 Centro de Investigación Biomédica en Red Enfermedades Respiratorias (CIBeRes); Infection and Immunity Program, Fundación Caubet-CIMERA, Carretera Soller km 2, 07110 Bunyola, Illes Balears, Spain; 5 Intensive Care Medicine Department, La Paz University Hospital, Paseo de la Castellana 261, 28046, Madrid, Spain

## Abstract

**Introduction:**

The use of glutamine as a dietary supplement is associated with a reduced risk of infection. We hypothesized that the underlying mechanism could be an increase in the expression and/or functionality of Toll-like receptors (TLR), key receptors sensing infections. The objective of this study was to evaluate whether glutamine supplementation alters the expression and functionality of TLR2 and TLR4 in circulating monocytes of trauma patients admitted to the intensive care unit (ICU).

**Methods:**

We designed a prospective, randomized and single-blind study. Twenty-three patients received parenteral nutrition (TPN) with a daily glutamine supplement of 0.35 g/kg. The control group (20 patients) received an isocaloric-isonitrogenated TPN. Blood samples were extracted before treatment, at 6 and 14 days. Expression of TLR2 and TLR4 was determined by flow cytometry. Monocytes were stimulated with TLR specific agonists and cytokines were measured in cell culture supernatants. Phagocytic ability of monocytes was also determined.

**Results:**

Basal characteristics were similar in both groups. Monocytes from patients treated with glutamine expressed the same TLR2 levels as controls before treatment (4.9 ± 3.5 rmfi vs. 4.3 ± 1.9 rmfi, respectively; *P *= 0.9), at Day 6 (3.8 ± 2.3 rmfi vs. 4.0 ± 1.7 rmfi, respectively; *P *= 0.7) and at Day 14 (4.1 ± 2.1 rfim vs. 4.6 ± 1.9 rmfi, respectively; *P *= 0.08). TLR4 levels were not significantly different between the groups before treatment: (1.1 ± 1 rmfi vs 0.9 ± 0.1 rmfi respectively; *P *= 0.9), at Day 6 (1.1 ± 1 rmfi vs. 0.7 ± 0.4 rmfi respectively; *P *= 0.1) and at Day 14 (1.4 ± 1.9 rmfi vs. 1.0 ± 0.6 rmfi respectively; *P *= 0.8). No differences in cell responses to TLR agonists were found between groups. TLR functionality studied by phagocytosis did not vary between groups.

**Conclusions:**

In trauma patients in the intensive care unit, TPN supplemented with glutamine does not improve the expression or the functionality of TLRs in peripheral blood monocytes.

**Trial registration:**

ClinicalTrials.gov Identifier: NCT01250080.

## Introduction

Glutamine is the most abundant nonessential amino acid in the human body. Besides its role as a constituent of proteins and its importance in amino acid transamination, glutamine may modulate immune cells [1]. Thus, glutamine deprivation reduces proliferation of lymphocytes, influences expression of surface activation markers of lymphocytes and monocytes, affects the production of cytokines, and stimulates apoptosis [1]. In addition, glutamine influences a variety of different molecular pathways. For example, glutamine stimulates the formation of heat shock protein 70 in monocytes by enhancing the stability of mRNA [2,3], influences the redox potential of the cell by enhancing the formation of glutathione [4,5], induces cellular anaerobic effects by increasing the cell volume [6,7], activates mitogen-activated protein kinases [8], and interacts with particular aminoacyl-transfer RNA synthetases in specific glutamine-sensing metabolism [2].

The innate immune system is the first line of host defence against pathogens and targets structurally conserved molecules, the so-called pathogen-associated molecular patterns (PAMPs) [9,10]. Innate responses are in most cases sufficient to eliminate invading microbes. Mammalian Toll-like receptors (TLR) comprise a family of germ line-encoded trans-membrane receptors which recognize PAMPs [9-11]. Activation of TLRs leads to the induction of inflammatory responses, phagocytosis but also to the development of antigen specific adaptive immunity [10]. Among this family of receptors, TLR2 and TLR4 have received great attention. TLR4 is essential for the recognition of lipopolysaccharide (LPS), a major component of Gram-negative bacteria, whereas TLR2 recognizes a large number of ligands including bacterial lipoteichoid acid and lipoproteins.

We and others [12-15] have shown that trauma patients present a dysregulation of the innate immune system, namely reduced expression of TLRs and blunted response to specific agonists markedly to LPS. Moreover, we have also shown that monocytes from trauma patients phagocytosized less efficiently than monocytes from control subjects [12]. On the other hand, clinical studies have shown that glutamine, as a dietary supplement for patients in critical condition, decreases the incidence of infection, primarily pneumonia, bacteremia, and sepsis [16,17]. It has been postulated, though not formally proven yet, that glutamine's beneficial effect could be due to a positive effect on the innate immune system. Given the importance of TLRs and TLRs-dependent signalling in host defence against infections we hypothesized that glutamine may increase the expression and/or functionality of TLRs, which in turn may have beneficial effects to clear infections. In a pilot report, in a general population of critical care patients, glutamine used as a dietary supplement did not increase the expression of TLR2 or TLR4 [18]. In this second report we have evaluated whether glutamine dietary supplement may affect not only the expression of TLR2 and TLR4 but also their functionality in circulating monocytes from peripheral blood in a specific group of trauma patients admitted to the ICU.

## Materials and methods

This prospective and comparative study took place at Son Dureta University Hospital (Palma de Mallorca, Spain), and was approved by the Ethics Committee of the Balearic Islands on 31 January 2007.

In all cases, informed consent for inclusion in the study was sought from the patient or the closest family member if the patient was unconscious.

### Study design

We designed a randomized, single blind, prospective study, with comparative therapeutic intervention with two groups: trauma patients treated with TPN supplemented with glutamine and those receiving TPN without glutamine.

Random selection was based on a computer-generated list that assigned patients to groups consecutively. Those who processed samples in the research unit did not know whether the patient had received glutamine or not.

### Patients and interventions

Trauma patients admitted to the intensive care unit (ICU) at a university third level hospital between 18 and 75 years (inclusive) with moderate to severe trauma, as defined by an Injury Severity Score (ISS) > 12 points were included in the study. Exclusion criteria were: patients who were under 17 and over 76 years of age, patients whose life expectancy was less than five days, who were allergic to glutamine, whose basic pathology included any serious immune system condition (diabetes, HIV, lupus, and so on) or who, in their long-term treatment prior to admission to ICU, received corticoids or any other immunosuppressant medication. A negative pregnancy test was required before women of childbearing age could be included in the study.

All patients received standardized advanced trauma life support (ATLS)-adapted emergency department treatment and standardized intensive care unit therapy.

All patients who were admitted to the ICU and received TPN as part of their treatment were selected for inclusion in the study. Indications for TPN treatment were based on the guidelines of the American Society of Parenteral and Enteral Nutrition (ASPEN) [19]. The indications for TPN were: contraindication for enteral nutrition (mainly abdominal surgery or abdominal trauma) or failure in achieve nutritional goals with enteral nutrition.

Of 43 consecutive patients who met the inclusion criteria, 23 were randomly assigned to receive a daily glutamine supplement of 0.35 g/kg weight as N2-L-Alanyl-L-Glutamine (0.5 g/kg/d - Dipeptiven Fresenius Kabi España) during five days. The treatment period of five days was chosen according to other clinical studies [16,20,21]. Basic TPN support for both groups was identical: StructoKabiven (Fresenius Kabi España), with a caloric intake of 28 kcal kg^-1 ^d^-1 ^and the following distribution of macronutrients: 0.28 g kg^-1 ^d^-1 ^of nitrogen, 3.5 g kg^-1 ^d^-1 ^of glucose and 1.08 g kg^-1 ^d^-1 ^of lipids, in addition to standard vitamins and trace elements. The control group (*n *= 20 patients) received a supplemental volume of the basic TPN solution to achieve an isocaloric and isonitrogenated formula with the study group. The total duration of the TPN, once the supplement with glutamine was finished after the fifth day, was based on clinical data and was decided by the clinician responsible for the patient.

Besides our previous study [18] screening the literature, we found no previous studies identifying a correlation between TLR and glutamine in humans. Therefore, it was determined that a sample size of 40 patients would be sufficient for this study.

In both groups, the peripheral blood samples for the study of TLRs in monocytes were extracted before beginning treatment (basal sample), at the end of the glutamine supplement (Day 6), and at 14 days ± 24 hours after initiating treatment.

These time points were chosen because the median length of stay of the trauma patients in our ICU is 10 days, which is in accordance with the data obtained from the ENVIN-HELICS study in Spain [22].

Because of the small volume of blood collected we could not perform all the analysis for each patient and, therefore, the phagocytosis assays were performed only for a small group of them. However, patients were not selected and were included consecutively as the different parts of the study were performed. The patients enrolled in the different sets of assays were homogenous in terms of severity and age.

### Data collection

Epidemiological data were collected, including date and time of sample extraction, description of the event that motivated ICU admission (diagnosis and severity scores), comorbidities of each patient and the appearance of any complications during ICU stay including total days of mechanical ventilation, ICU and hospital length of stay.

Among the data collected there were all the treatments that patients received during their ICU stay, especially all pharmacological treatments with known anti-inflammatory properties that could affect the study results. All members of both of the two patient groups were handled and treated equivalently.

With respect to infections, samples were analyzed whenever there was a clinical suspicion of possible infection [23]. The definition of nosocomial infection used in this study is that proposed by the CDC [24] and it was mainly based on microbiological findings. Blood and other cultures were done at our institution following standard microbiological procedures, including incubation in anaerobic atmosphere when applicable [25].

### Flow cytometry

Expression of TLR2 and TLR4 in peripheral blood monocytes was determined by flow cytometry. Blood samples (one sample per patient) were collected in a K2-anticoagulation medium. It is known that this medium does not affect the expression of TLR2 and TLR4 [26]. A total of 100 μL was incubated with a combination of anti-CD14 fluorescein conjugated (clone My4, 10 μg/mL; Beckman Coulter, Brea, California, USA) and anti-TLR2 (clone TL2.1, 10 μg/mL; ebioscience, San Diego, California, USA) or anti-TLR4 (clone HTA125, 10 μg/mL; ebioscience, San Diego, California, USA) phycoerythrin conjugated in the presence of 25 μL of fetal calf serum during 30 minutes at 4°C. A total of 2 ml of FACS lysing solution (Beckton Dickinson, Franklin Lakes, New Jersey, USA) was added to the samples which were incubated 10 minutes at room temperature. Samples were centrifuged in a clinical centrifuge (530 *× g*, 5 minutes, 25°C) and the cellular pellet was washed once with 1% BSA-0.1% sodium azide in PBS. Finally cells were resuspended in 500 μl IsoFlowTM Sheath Fluid (Beckman Coulter). The analyses were carried out in an Epics XL flow cytometer using the Expo32 software (Beckman Coulter, Brea, California, USA). Monocytes were identified by gating on a side versus CD14 dot plot. The levels of TLR2 and TLR4 were expressed as relative mean fluorescence intensity (r*mfi*). The non-specific binding was corrected by subtraction of *mfi *values corresponding to isotype matched antibodies. A total of 10,000 monocytes were analysed in every experiment.

### Monocyte isolation and stimulation

Blood samples collected in 3.8% sodium citrate tubes, were diluted 1:5 in RPMI-1640 supplemented with 10% heat inactivated Fetal Calf Serum (FCS), glutamine (2 mM), HEPES (200 mM) and antibiotics (penicillin-streptomycin) and monocytes were obtained using a commercial isolation kit exactly as recommended by the manufacturer (Dynal monocyte negative isolation kit, Oxoid, Cambridge, United Kingdom). This collection method does not affect TLR-ligand induced cytokine response [26]. Lymphocytes represent less than 5% of the cells after this procedure. Cell viability was assessed by trypan blue dye exclusion and was > 95%. Cells were finally resuspended at a cell density of 10^6 ^cells/ml in RPMI-1640 medium supplemented with 10% heat inactivated FCS, glutamine (2 mM), HEPES (200 mM) and antibiotics (penicillin-streptomycin). Cells were cultured in 96-well plates at a cell density of 10^5 ^per well. Cells were stimulated with different amounts of purified LPS from *Escherichia coli *O111:B4 (Sigma Chemicals, Saint Louis, Missouri, USA), Pam3CSK4 (PAM; Invivogen, San Diego, California, USA) or zymosan (Invivogen). LPS was repurified exactly as previously described [27]. This procedure results in LPS preparations that utilize TLR4, and not TLR2, for signalling. After 16 hours cell culture supernatants were collected, cell debris was removed by centrifugation, and samples were frozen at -80°C until assayed.

### Cytokine analysis

We determined the concentration of IL-1β, IL-6, TNFα and IL-10 in cell culture supernatants using a bead array ELISA according to the instructions of the manufacturer (CBA Kit, BD Biosciences, Franklin Lakes, New Jersey, USA). The assay sensitivity for each cytokine was 7.2 pg/mL for IL-1β, 2.5 pg/mL for IL-6, 3.7 pg/mL for TNFα and 3.3 pg/mL for IL-10.

### Phagocytosis

To determine the phagocytic capability of monocytes, the assay described by Blander *et al*. was performed [28]. Briefly, live *Escherichia coli *expressing green fluorescent protein was added to 100 μL of whole blood collected in K2-anticoagulation medium tubes. Bacteria were added at a ratio of 100 bacteria per monocyte. After 30-minutes incubation at 37°C, samples were centrifuged in a clinical centrifuge (530 *× g*, 5 minutes, 25°C) and the cellular pellet was washed once with 1% BSA-0.1% sodium azide in PBS. Finally cells were resuspended in 1 mL IsoFlowTM Sheath Fluid (Beckman Coulter). The analyses were carried out in an Epics XL flow cytometer using the Expo32 software. Monocytes were identified by gating on a side versus CD14 dot plot and GFP fluorescence recorded. Results were expressed as relative mean fluorescence intensity (r*mfi*) measured in arbitrary units after substraction of *mfi *values corresponding to monocytes labeled with CD14 antibody. A total of 10,000 monocytes were analysed in every experiment. Phagocytosis was performed in serum-free media to eliminate contributions of Fc and/or complement receptors.

### Statistical analysis

The quantitative variables are expressed as the mean and standard deviation (SD) or as the median and interquartiles. Qualitative variables are expressed as percentages, with a confidence interval of 95% (CI 95%). To determine whether variables followed a normal distribution or not, we used the Shapiro Wilks test.

For the comparison of quantitative variables in two independent samples the Student's *t*-test was used if the variable followed a normal distribution and the Mann-Whitney U-test in skewed samples. In more than two related samples, all of them were initially compared by the Friedman-test. Then differences in values were tested by pairwise comparisions using the Wilcoxon's signed rank test with Bonferroni's correction. For the comparison of qualitative variables, we used *chi*-square or Fisher's exact test, as necessary.

For all comparisons, we considered statistical significance to be a two-tailed alpha error probability of ≤ 5% (*P *≤ 0.05). Statistical analysis was performed by using SPSS version 15 (SPSS Inc., Chicago, IL, USA).

## Results

### Clinical data

From February 2007 through June 2008, 43 consecutive patients who met the inclusion criteria were randomly assigned to receive a TPN with a daily supplement of glutamine or not.

There were no statistically significant differences in basal characteristics of both groups of patients treated with and without glutamine (Table 1). Like some other investigators we did not observe any adverse effect, studied through the SOFA score, due to the use of these doses of glutamine (Table 1).

**Table 1:** Baseline characteristics of patient population

	TPN with Gl (*n *= 23)	TPN without Gl (*n *= 20)	*P*-value
Age (years)	34.2 ± 14.7	40.4 ± 15.2	0.18
Male/Female	19/4	18/2	0
Weight (Kg)	77.3 ± 11.3	81.9 ± 11.1	0.19
SAPS	35.8 ± 9.5	31.4 ± 13.5	0.27
APACHE 2	19.2 ± 3.2	15.1 ± 9.3	0.12
APACHE 3	48.3 ± 18.3	36.1 ± 18.3	0.06
ISS	31.4 ± 12.3	31.6 ± 12.6	0.96
Previous surgery	8	12	0.43
Previous shock	6	4	0.73
SOFA pretreatment	7 ± 3.7	7 ± 3	0.96
TPN beginning (days)	4.7 ± 3.1	4.3 ± 2.1	0.67
TPN duration	14 (8 to 19)	14.5 (8 to 23)	0.43
Norepinephrine	0.05 ± 0.1	0.2 ± 0.6	0.44
Pretreat. infection	11	9	0.98
SOFA postreatment	6.3 ± 3.4	6.8 ± 4.4	0.69

Data are presented as mean ± SD; number of patients or median (25th to 75th percentile).

SAPS, Simplified Acute Physiology Score; APACHE, Acute Physiology and Chronic Health Evaluation; ISS, Injury Severity Score; Previous surgery, number of patients that required surgery before randomization; Previous shock, number of patients who presented a hemorrhagic shock before randomization; SOFA pretreatment, Sequential Organ Failure Assessment before treatment; TPN beginning, Number of days since hospital admission before the patients were included in the study; TPN duration, Total duration of the TPN in days; Norepinephrine, Medium dose of norepinephrine in μg × Kg^-1 ^× minute^-1 ^during the five days of the treatment; Pretreat infection, Number of patients with an infection before the randomization; SOFA postreatment, Sequential Organ Failure Assessment after treatment (Day 6).

There were detected 21 positive cultures in the group of patients treated with glutamine and 32 positive cultures in the control group (Table 2). The median of ICU length of stay was similar in both groups and there was a trend in the median of the hospital length of stay not reaching statistically significance (Table 2).

**Table 2:** Complications and outcome of patients.

	TPN with Gl (*n *= 23)	TPN without Gl (*n *= 20)	*P*-value
Infections, *n *(%)			
Respiratory infection	14 (61%)	14 (70%)	0.53
Urinary infection	1 (4%)	2 (10%)	0.6
Blood culture	1 (4%)	5 (25%)	0.08
Catheter infection	4 (17%)	6 (30%)	0.5
CSF infection	1 (4%)	1 (5%)	0.6
Wound infection	0 (0%)	4 (20%)	0.08
Pneumonia	11 (48%)	8 (40%)	0.6
Length of MV (days)	15.2 ± 8.2	18.9 ± 11.1	0.21
ICU length of stay (days)	21 (17 to 25)	21 (14 to 47)	0.47
Hospital length of stay (days)	31 (19 to 42)	40 (24 to 80)	0.23
ICU mortality	4 (17%)	2 (10%)	0.7
Hospital mortality	0 (0%)	1 (5%)	1

Data are presented as mean±SD or median (25th to 75th percentile).

Respiratory infection, number of positive bronchial aspirate cultures during ICU admission; Urinary infection, number of positive urine cultures during ICU admission; Blood culture, number of positive blood cultures during ICU admission; Catheter infection, number of positive blood cultures during ICU admission; CSF infection, number of positive cultures of Cerebro Spinal Fluid; Wound infection, number of positive cultures in the wound zone; Pneumonia, number of patients who developed nosocomial pneumonia during ICU admission; Length of MV, number of days of mechanical ventilation.

### Surface expression of TLR2 and TLR4

**Figure 1:**
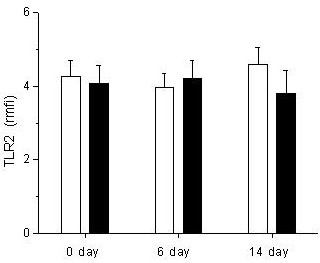
**Expression of TLR2 in trauma patients treated with and without glutamine**. The expression of TLR2 was analyzed in CD14 positive peripheral blood mononuclear cells. r*mfi *are shown for 23 trauma patients treated with glutamine (black bars) and 20 trauma patients without glutamine and used as controls (white bars). Samples were obtained at the beginning of the treatment (Day 0); at the end of the treatment (Day 6) and at Day 14. Data are given as mean ± SEM.

Monocytes from patients treated with glutamine expressed the same TLR2 levels than monocytes from control subjects before treatment (4.9 ± 3.5 rmfi vs. 4.3 ± 1.9 rmfi, respectively; *P *= 0.9), at Day 6 (3.8 ± 2.3 rmfi vs. 4 ± 1.7 rmfi, respectively; *P *= 0.7) and at Day 14 (4.1 ± 2.1 rfim vs. 4.6 ± 1.9 rmfi, respectively; *P *= 0.08) (Figure 1).

**Figure 2:**
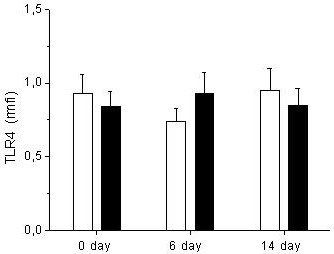
**Expression of TLR4 in trauma patients treated with and without glutamine**. The expression of TLR4 was analyzed in CD14 positive peripheral blood mononuclear cells. r*mfi *are shown for 23 trauma patients treated with glutamine (black bars) and 20 trauma patients without glutamine and used as controls (white bars). Samples were obtained at the beginning of the treatment (Day 0); at the end of the treatment (Day 6) and at Day 14. Data are given as mean ± SEM.

Concerning TLR4 expression, monocytes from patients who received glutamine supplementation also expressed similar levels of TLR4 than monocytes from the control group before treatment (1.1 ± 1 rmfi vs 0.9 ± 0.1 rmfi respectively; *P *= 0.9), at Day 6 (1.1 ± 1 rmfi vs. 0.7 ± 0.4 rmfi respectively; *P *= 0.1) and at Day 14 (1.4 ± 1.9 rmfi vs. 1 ± 0.6 rmfi respectively; *P *= 0.8) (Figure 2).

### TLR functionality

Stimulation of monocytes with TLR specific agonists is assumed as a marker for immune response *in vivo *[26]. We asked whether a glutamine dietary supplement may affect the response of monocytes to different TLR agonists. To this end, we measured the levels of TNFα, IL-1β, IL-6 and IL-10 in supernatants of monocytes challenged with either LPS (100 ng/mL), TLR4 agonist, Pam3CSK4 (10 μg/mL) or zymosan (10 μg/mL), two TLR2 agonists.

**Figure 3:**
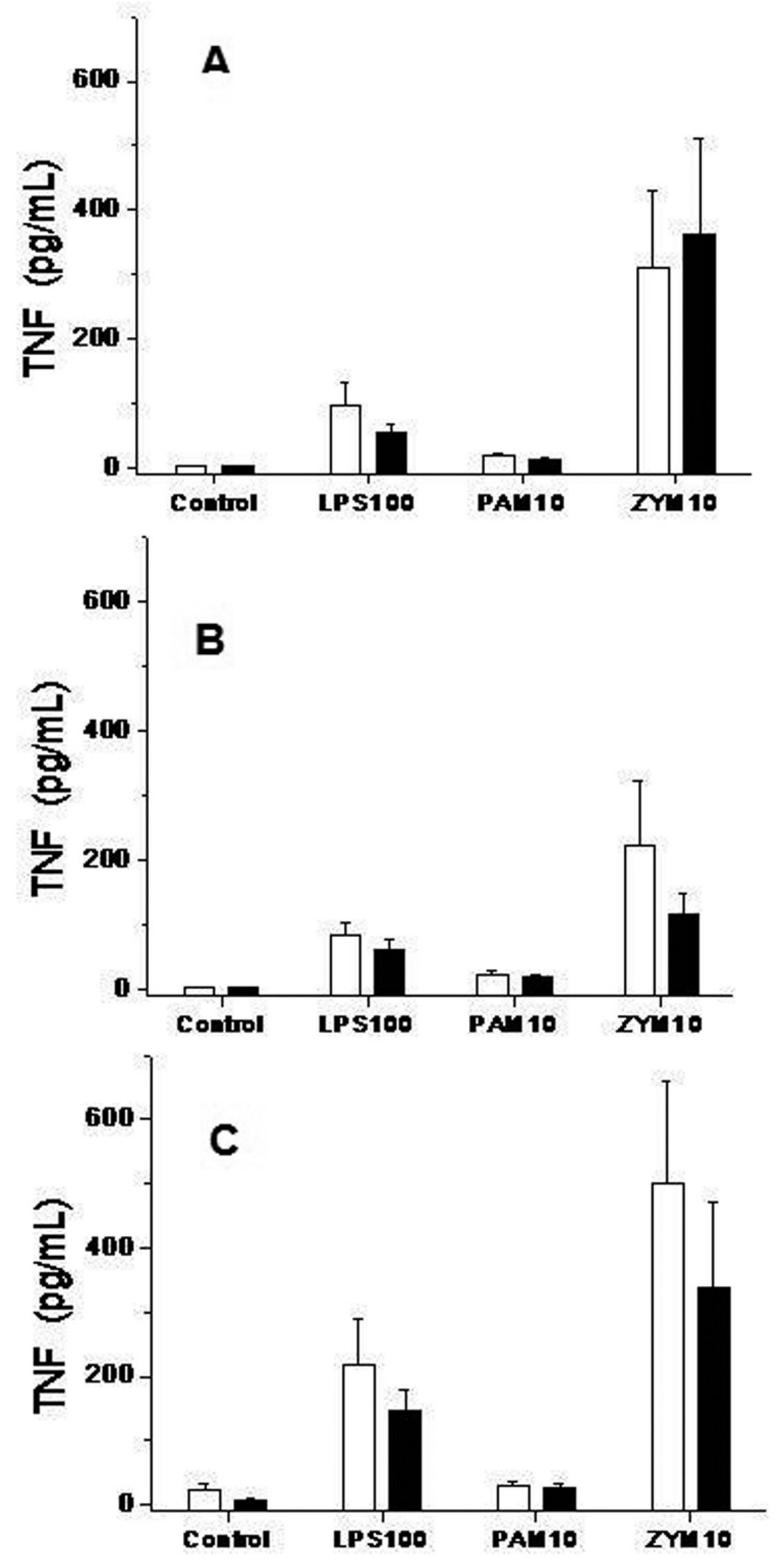
**Concentration of TNFα in cell culture supernatants in trauma patients treated with and without glutamine**. TLR functionality. Levels of TNFα analyzed by a bead array ELISA (CBA Kit, BD Biosciences), in response to lipopolysaccharide (LPS-100 ng/mL), Pam3CSK4 (PAM-10 pg/mL) and zymosan (ZYM-10 pg/mL) at the beginning of the treatment (Figure 3A); at Day 6 (Figure 3B) and at Day 14 (Figure 3C). Monocytes from trauma patients treated with glutamine subjects (black bars, *n *= 23) and trauma patients without glutamine (white bars, *n *= 20). Control bars are samples production of cytokines by unstimulated monocytes. Data are given as mean ± SEM.

**Figure 4:**
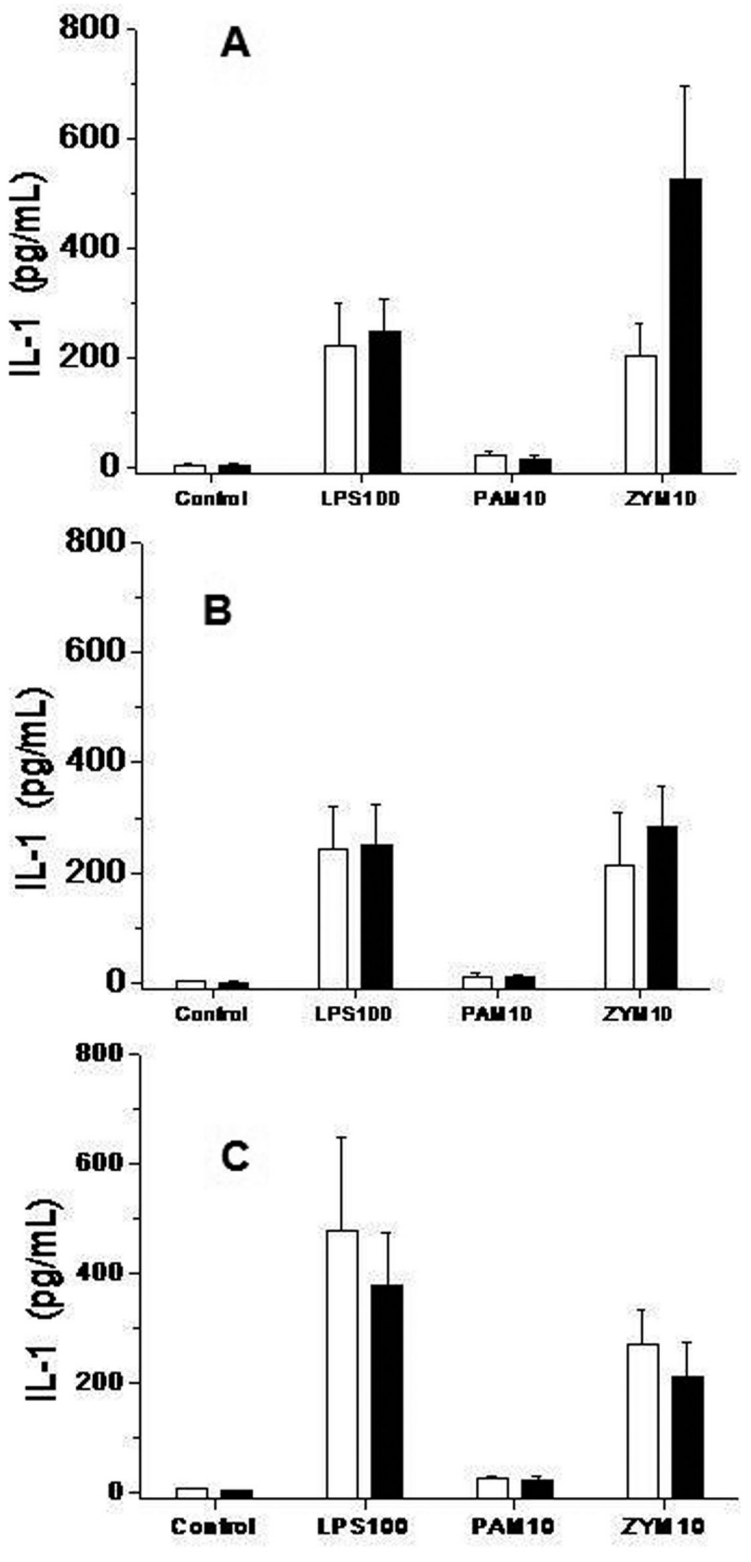
**Concentration of IL1β in cell culture supernatants in trauma patients treated with and without glutamine**. TLR functionality. Levels of IL1β analyzed by a bead array ELISA (CBA Kit, BD Biosciences), in response to lipopolysaccharide (LPS-100 ng/ml), Pam3CSK4 (PAM-10 pg/mL) and zymosan (ZYM-10 pg/mL) at the beginning of the treatment (Figure 4A); at Day 6 (Figure 4B) and at Day 14 (Figure 4C). Monocytes from trauma patients treated with glutamine subjects (black bars, *n *= 23) and trauma patients without glutamine (white bars, *n *= 20). Control bars are samples production of cytokines by unstimulated monocytes. Data are given as mean ± SEM.

**Figure 5:**
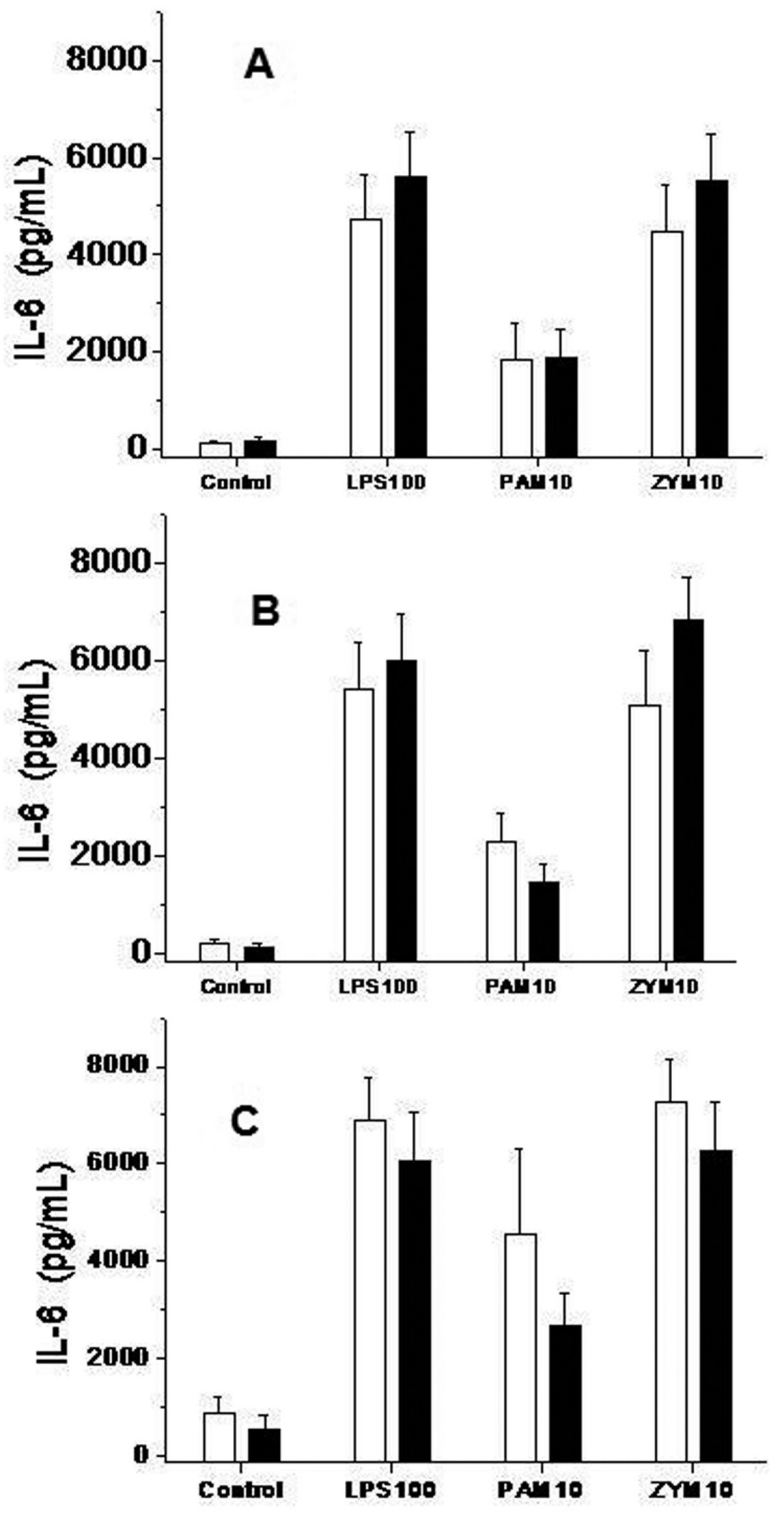
**Concentration of IL6 in cell culture supernatants in trauma patients treated with and without glutamine**. TLR functionality. Levels of Cytokines IL 6 analyzed by a bead array ELISA (CBA Kit, BD Biosciences), in response to lipopolysaccharide (LPS-100 ng/ml), Pam3CSK4 (PAM-10 pg/mL) and zymosan (ZYM-10 pg/mL)) at the beginning of the treatment (Figure 5A); at Day 6 (Figure 5B) and at Day 14 (Figure 5C). Monocytes from trauma patients treated with glutamine subjects (black bars, *n *= 23) and trauma patients without glutamine (white bars, *n *= 20). Control bars are samples production of cytokines by unstimulated monocytes. Data are given as mean ± SEM.

**Figure 6:**
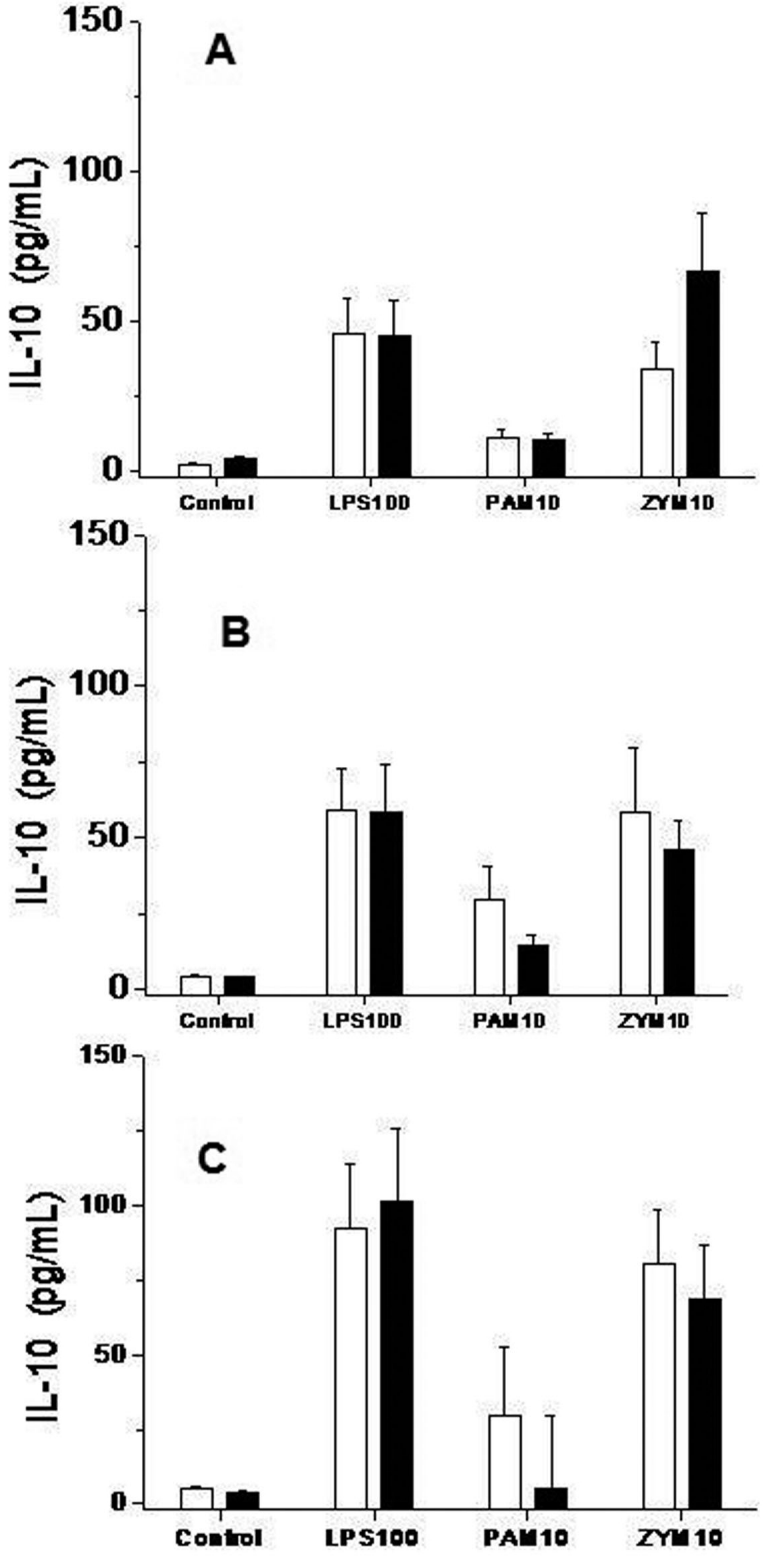
**Concentration of IL10 in cell culture supernatants in trauma patients treated with and without glutamine**. TLR functionality. Levels of Cytokines IL 10 analyzed by a bead array ELISA (CBA Kit, BD Biosciences), in response to lipopolysaccharide (LPS-100 ng/ml), Pam3CSK4 (PAM-10 pg/mL) and zymosan (ZYM-10 pg/mL)) at the beginning of the treatment (Figure 6A); at Day 6 (Figure 6B) and at Day 14 (Figure 6C). Monocytes from trauma patients treated with glutamine subjects (black bars, *n *= 23) and trauma patients without glutamine (white bars, *n *= 20). Control bars are samples production of cytokines by unstimulated monocytes. Data are given as mean ± SEM.

We present the results of the stimuli that induced the strongest response. The levels of TNFα (Figure 3), IL-1β (Figure 4), IL-6 (Figure 5) and IL-10 (Figure 6) produced in response to LPS, Pam3CSK4 or zymosan were similar in patients treated with and without glutamine pretreatment, at Day 6 and at Day 14.

We also performed dose-response experiments using lower concentrations of the same agonists and we only found differences in the production of IL-10 after stimulation with zymosan 0.1 μg/mL at baseline level (3.8 pg/dL in the glutamine group vs 2 pg/dL in the control group) and in the production of IL-1β at Day 14 after Pam3CSK4 with 1 μg/mL stimulation (12.8 pg/dL in the glutamine group vs 16.9 pg/dL in the control group). For the rest of the 106 comparisons between both groups and the different dose-response experiments, no statistically significant differences were found.

We also asked whether glutamine dietary supplement could alter the responses of monocytes for the three agonists at the three time points studied (baseline, Day 6 and Day 14) for each patient receiving the treatment. For this purpose and because there were more than two related samples, all of them were initially compared by the Friedman-test. Then differences in values were tested by pairwise comparisions using the Wilcoxon's signed rank sum test with Bonferroni's correction. Within the group of patients who received glutamine we found an increase in the production of TNFα after stimulation with LPS 100 ng/mL (55.2 pg/dL at baseline; 63 pg/dL at Day 6; 146 pg/dL at Day 14), the production of IL-10 after stimulation with LPS 100 ng/mL (45 pg/dL at baseline, 58 pg/dL at Day 5, 101 pg/dL at Day 14), the production of IL-6 after LPS 100 ng/mL stimulation (5591 pg/dL at baseline; 6004 pg/dL at Day 6; 6065 pg/dL at Day 14) and the production of IL-1β after LPS 100 ng/mL (249 pg/dL at baseline; 253 pg/dL at Day 6; 379 pg/dL at Day 14). The rest of the stimulations with Pam3CSK4 and zymosan at different doses did not vary significantly over time in the group of patients treated with glutamine.

However, we also found an increase in the cellular responses to LPS over time in monocytes from the control group. Thus, levels of TNFα in supernatants of LPS-treated monocytes were higher at Day 14 than at Day 6 or baseline (96 pg/dL at baseline; 84 pg/dL at Day 6, 218 pg/dL at Day 14). Likewise, levels of IL-10 after stimulation were also higher at Day 14 than at baseline (45 pg/dL at baseline; 59 pg/dl at Day 6; 92 pg/dL at Day 14). Like in the group of patients treated with glutamine, the rest of stimulations with Pam3CSK4 and zymosan at different doses did not affect significantly over time.

### Phagocytosis

Phagocytosis of pathogens also relies on the activation of TLRs [28]. The phagocytic capability of both groups studied before the beginning of the treatment, or at the end of the treatment (Day 6) or at Day 14 was not significantly different at any time point studied (Table 3).

**Table 3:** Phagocytosis capability in patients treated with and without glutamine

	TPN with Gl (*n *= 18)	TPN without Gl (*n *= 14)	*P*-value
Pretreatment	61.3 ± 20.8	58.8 ± 24.6	0.8
Day 6	50.2 ± 22.8	51.8 ± 9	0.8
Day 14	56.5 ± 25.3	55.1 ± 21.5	0.9

Results were expressed as relative mean fluorescence intensity (r*mfi*). Data are presented as mean ± SD.

## Discussion

In this study we have shown that the TLR dysregulation previously found in trauma ICU patients, reduced levels of TLR2 and TLR4 expression, blunted response to TLR agonists and reduced phagocytic ability of monocytes, cannot be alleviated by glutamine dietary supplement.

One meta-analysis [29] reviewed seven studies with 326 cases that included a complication of infection, and found a significant reduction in the number of infections in the group of patients treated with glutamine: RR 0.80; CI 95%; 0.64 to 1.00; *P *= 0.03. In addition recent ESPEN guidelines recommend the use of glutamine when TPN is indicated in ICU patients [30]. In our study, the treatment group also presented a reduced incidence of infections and a reduced hospital length of stay, although neither finding achieved statistical significance. In any case, our study was not designed to test the clinical efficacy of glutamine for a significant reduction of the number of infections and/or hospital length of stay, so this limitation precludes any conclusion about efficacy.

The possible beneficial effects of glutamine on the functionality of the innate immune system are poorly characterized although these effects might be the underlying explanation of glutamine clinical effect on reducing infectious complications. Taking into account that TLRs play a central role in the activation of the innate system, hence leading to the activation of different intracellular signalling cascades involved in the activation of host defence mechanisms, in this study we focused on the effect of glutamine on the expression and functionality of TLR2 and TLR4. A wealth of evidence indicates that these TLRs recognize a plethora of pathogens. In fact, a recent experimental study, treatment with enteral glutamine was associated with down-regulation of TLR-4, MyD88 and TRAF6 expression and concomitant decrease in intestinal mucosal injury caused by LPS endotoxaemia in rats [31]. These authors conclude that the positive effect of glutamine on intestinal structure after LPS endotoxaemia may be considered as a mechanism via which immunonutrition helps in the recovery of critically ill patients.

As a population studied, we chose trauma patients admitted to the ICU for various reasons. First, in a previous study [12] we did demonstrate that the TLR expression and functionality are altered in monocytes from traumatic patients, and that this alteration persists during the first 14 days after hospital admission. Second, several studies have demonstrated that a decrease or even total lack of TLR expression correlate with greater susceptibility to infection [32-34]. Altogether, trauma patients make a good case study to test whether glutamine dietary supplement may improve TLR-dependent host defence mechanisms. On the other hand, it seems reasonable to think that if we could improve TLR-dependent host defence mechanisms by using a pharmaconutrient such as glutamine the molecular mechanisms to detect microorganisms might improve, resulting in a reduced incidence of infectious complications. However, the results of this study show that the TPN supplemented with glutamine does not change the expressions of TLR2 or TLR4, the secretion of cytokines upon stimulation with TLR agonists and the phagocytic capability. Nevertheless critical care patients are heterogeneous and it is possible that a hyperinflammatory response coexists with a dysfunction in the immune system. As it has been previously pointed out, TLR-4 expression is lower in trauma patients than in healthy volunteers [12,13] whereas in septic patients TLR expression increased [35,36].

In general it is assumed that the levels of TLRs correlate with the cellular response upon stimulation with specific agonists [26]. For example, macrophages overexpressing TLRs, release higher amounts of inflammatory mediators upon TLR engagement [37,38]. It is also known that cells from trauma patients secrete significantly less inflammatory cytokines than cells from control subjects when LPS, a TLR4 agonist, is used [12,13,39,40]. However, our data show that cells from trauma patients treated with glutamine secreted similar amounts of cytokines than cells from control subjects upon stimulation with TLR2 and TLR4 agonists.

It is also known that phagocytosis is impaired in monocytes from trauma patients [12]. Phagocytosis is an ancient form of host defence which is dependent on several signalling pathways including TLR-dependent signals [28]. Thus, it has been shown that activation of the TLR signalling by bacteria regulates phagocytosis at multiple steps, including internalization and phagosome maturation [28]. Nevertheless, our findings, likewise previous ones in paediatric patients [41], show that glutamine supplementation dose not increase the phagocytic capacity.

### Limitations of the study

It must be commented that there is controversy over the surface expression of TLR2 and TLR4 by leukocytes from traumatic patients. In our previous work [12], we showed a reduced expression of both TLR2 and TLR4 in monocytes from those trauma patients who developed any infection. On the other hand, Adib-Conquy *et al*. [13] reported a reduced expression of TLR4 in severely injured patients early after trauma, whereas TLR2 remained unchanged. In contrast, another study [15] showed a down-regulation of the expression of both TLR2 and TLR4, whereas Lendemans *et al*. [14] observed a decrease of only TLR2 expression. Differences in the patients analyzed may account for these conflicting results and we can not rigorously rule out that technical issues such as the commercial source of the antibodies used or the way the cells were fixed for the flow cytometry experiments may also be responsible for these conflicting results.

It also should be pointed out that an *in vivo *scenario is quite complex and the final outcome of an infectious process depends on the concerted action of several cells, including epithelial, endothelial, neutrophils, macrophages and lymphocytes, and therefore, we cannot rule out that glutamine may exert a positive effect on other cell types or even at the level of cross-talk between cells of the innate immune system. Studies are on going to test these hypotheses.

In this study, we have analyzed different phenotypes of circulating cells over time. It should be taken into consideration that initial phenotypes may be compensated after three to five days owing to the influx of new and immature monocytes. In fact, this might be the explanation underlying the increased response to different agonists after six days. In any case, our data suggest that glutamine dietary supplement may not affect cell turnover since the increased response was found in both groups and, furthermore, no significant differences were found between them.

Another limitation of the study is that we did not measure plasma levels of free glutamine. Nevertheless it must be said that previous studies have documented low levels of glutamine in previously fit trauma patients, and that the dose of glutamine employed in our study and the length of treatment was enough to correct any deficiency. It also should be noted that for the reported analysis of TLR expression and phagocytic ability, whole blood samples, without subculturing cells, were used. However, for the stimulation experiments using different TLR agonists purified monocytes were challenged with stimuli in tissue culture medium containing glutamine which is commonly used to culture cells and perhaps this glutamine present in the medium may mask differences between experimental groups. Nevertheless, the impaired LPS response displayed by monocytes from trauma patients reported by us and others [12-15] was still found in both groups.

## Conclusions

The results of this study in trauma ICU patients show that TPN supplemented with glutamine does neither improve the expression of TLR-2 or TLR-4 in circulating monocytes from peripheral blood, nor the functionality of TLR-2 and TLR-4 studied by analyzing the cytokine production after monocyte isolation and stimulation or by studying the phagocytic capability.

## Key messages

• The use of glutamine as a dietary supplement is associated with a reduced risk of infection. It has been postulated, though not formally proven yet, that glutamine beneficial effect could be due to a positive effect on the innate immune system.

• Given the importance of TLRs and TLRs-dependent signalling in host defence against infections we hypothesized that glutamine may increase the expression and/or functionality of TLRs, which in turn may have beneficial effects to clear infections.

• Nevertheless, the results of this study show that the TPN supplemented with glutamine does neither improve the expression of TLR-2 or TLR-4 in circulating monocytes from peripheral blood, nor the functionality of TLR-2 and TLR-4 studied by analyzing the cytokine production after monocyte isolation and stimulation or by studying the phagocytic capability.

## Abbreviations

ASPEN: American Society of Parenteral and Enteral Nutrition; ATLS: advanced trauma life support; FCS: fetal calf serum; FITC: fluorescein; ICU: intensive care unit; IL: interleukin; ISS: Injury Severity Score; LPS: lipopolycaccharide, mfi: mean fluorescence intensity; PAMPs: pathogen associated molecular patterns; PE: ficoeritrin; SOFA: Sepsis related Organ-Failure Assessment; TLR: toll-like receptors; TNF: tumour necrosis factor; TPN: parenteral nutrition.

## Competing interests

This work was funded by a grant from the ESPEN Peter Furst Research Prize awarded to JPB. All other authors declare that they have no competing interests.

## Authors' contributions

JPB assisted with design, analysis and interpretation of data, and writing the article. CC and VR assisted with flow cytometry. PM and JMR assisted with design, analysis, and writing the article. JI gave final approval to the version to be published. AGLM revised the article critically and gave final approval to the version to be published. JAB assisted with flow cytometry and analysis of data. All authors read and approved the final manuscript.

## References

[B1] Roth E Nonnutritive effects of glutamine J Nutr 2008 138 2025S 2031S 18806119 10.1093/jn/138.10.2025S

[B2] Eliasen MM Brabec M Gerner C Pollheimer J Auer H Zellner M Weingartmann G Garo F Roth E Oehler R Reduced stress tolerance of glutamine-deprived human monocytic cells is associated with selective down-regulation of Hsp70 by decreased mRNA stability J Mol Med 2006 84 147 158 10.1007/s00109-005-0004-6 16308684

[B3] Singleton KD Wischmeyer PE Glutamine's protection against sepsis and lung injury is dependent on heat shock protein 70 expression Am J Physiol Regul Integr Comp Physiol 2007 292 R1839 1845 17234954 10.1152/ajpregu.00755.2006

[B4] Roth E Oehler R Manhart N Exner R Wessner B Strasser E Spittler A Regulative potential of glutamine-relation to gluthatione metabolism Nutrition 2002 18 217 221 10.1016/S0899-9007(01)00797-3 11882392

[B5] Hong RW Rounds JD Helton WS Robinson MK Wilmore DK Glutamine preserves liver glutathione after lethal hepatic injury Ann Surg 1992 215 114 119 10.1097/00000658-199202000-00004 1546897 PMC1242397

[B6] Haussinger D Roth E Lang F Gerok W Cellular hydratation state: an important determinant of protein catabolism in health and disease Lancet 1993 341 1330 1332 10.1016/0140-6736(93)90828-5 8098459

[B7] Oehler R Zellner M Hefel B Weingartmann G Spittler A Struse HM Roth E Influence of heat shock protein on cell volume regulation: protection from hypertonic challenge in a human monocyte cell line FASEB J 1998 12 553 560 9576482 10.1096/fasebj.12.7.553

[B8] Eliasen MM Winkler W Jordan V Pokar M Marchetti M Roth E Allmaier G Oehler R Adaptative cellular mechanisms in response to glutamine starvation Front Biosci 2006 11 3199 3211 10.2741/2043 16720386

[B9] Janeway CA Jr Medzhitoz R Innate immune recognition Annu Rev Immunol 2002 20 197 216 10.1146/annurev.immunol.20.083001.084359 11861602

[B10] Akira S Takeda K Kaisho T Toll-like receptors: critical proteins linking innate and acquired immunity Nat Immunol 2001 2 675 680 10.1038/90609 11477402

[B11] Van Amersfoort ES Van Berkel TJC Kuiper J Receptors, mediators, and mechanisms involved in bacterial sepsis and septic shock Clin Microbiol Rev 2003 16 379 414 10.1128/CMR.16.3.379-414.2003 12857774 PMC164216

[B12] Pérez-Bárcena J Regueiro V Crespí C Pierola J Oliver A Llompart-Pou JA Ayestarán JI Raurich JM Marsé P Ibáñez J Bengoechea JA Expression of Toll-Like Receptors 2 and 3 is upregulated during hospital admission in traumatic patients. Lack of correlation with blunted innate immune responses Ann Surg 2010 251 521 527 20134316 10.1097/SLA.0b013e3181cc8f84

[B13] Adib-Conquy M Moine P Asehnoune K Edouard A Espevik T Miyake K Werts C Cavaillon JM Toll-like receptor mediated tumor necrsosis factor and interleukin-10 production differ during systemic inflammation Am J Respir Crit Care Med 2003 168 158 164 10.1164/rccm.200209-1077OC 12738604

[B14] Lendemans S Kreuzfelder E Rani M Bayeeh E Schade FU Flohé SB Waydhas C Flohé S Toll-like receptor 2 and 4 expression after severe injury is not involved in the dysregulation of the innate immune system J Trauma 2007 63 740 746 10.1097/01.ta.0000240451.42238.d1 18089999

[B15] Laudanski K De A Brouxhon S Kyrkanides S Miller-Graziano C Abnormal PGE (2) regulation of monocyte TNF-alpha levels in trauma patients parallels development of a more macrophage-like phenotype Shock 2004 22 204 212 10.1097/01.shk.0000135289.62159.ad 15316388

[B16] Déchelotte P Hasselmann M Cynober L Allaouchiche B Coëffier M Hecketsweiler B Merle V Mazerolles M Samba D Guillou YM Petit J Mansoor O Colas G Cohendy R Barnoud D Czernichow P Bleichner G L-alanyl-L-Glutamine dipeptide supplemented total parenteral nutrition reduces infectious complications and glucose intolerance in critically ill patients: The French controlled, randomized, double-blind, multicenter study Crit Care Med 2006 34 598 604 16505644 10.1097/01.CCM.0000201004.30750.D1

[B17] Griffiths RD Allen KD Andrews FJ Jones C Infection, multiple organ failure, and survival in the intensive care unit: influence of glutamine-supplemented parenteral nutrition on acquired infection Nutrition 2002 18 546 552 10.1016/S0899-9007(02)00817-1 12093428

[B18] Pérez-Bárcena J Regueiro V Marsé P Raurich JM Rodríguez A Ibáñez J de Lorenzo Mateos AG Bengoechea JA Glutamine as a modulator of the immune system of critical care patients: effect on Toll-Like receptor expression. A preliminary study Nutrition 2008 24 522 527 18367379 10.1016/j.nut.2008.01.056

[B19] ASPEN Board of Directors and the Clinical Guidelines TASK Force Guidelines for the use of enteral and parenteral nutrition in adult and pediatric patients JPEN J Parenter Enteral Nutr 2002 26 1sa 138sa 10.1177/0148607102026001011 11841046

[B20] Goeters C Wenn A Mertes N Wempe C Van Aken H Stehle P Bone HG Parenteral L-alanyl-L-glutamine improves 6-month outcome in critically ill patients Crit Care Med 2002 30 2032 2037 10.1097/00003246-200209000-00013 12352037

[B21] Mertes N Schulzki C Goeters C Winde G Benzing S Kuhn KS Van Aken H Stehle P Fürst P Cost containment though L-alanyl-L-Glutamine supplemented total parenteral nutrition after major abdominal surgery: a prospective randomized double-blind controlled study Clin Nutr 2000 19 395 401 10.1054/clnu.2000.0142 11104589

[B22] Alvarez-Lerma F Palomar M Olaechea P Otal JJ Insausti J Cerdá E Grupo de Estudio de Vigilacia de Infección Nosocomial en UCI National Study of Control of Nosocomial Infection in Intensive Care Units. Evolutive report of the years 2003-2005 Med Intensiva 2007 31 6 17 10.1016/S0210-5691(07)74764-2 17306135

[B23] Guidelines for the management of severe sepsis and septic shock. The International Sepsis Forum Intensive Care Med 2001 27 S1 S134 10.1007/s001340000767 11519475

[B24] Garner JS Jarvis WR Emori TG Horan TC Hughes JM CDC definitions for nosocomial infections Am J Infect Control 1988 16 128 140 10.1016/0196-6553(88)90053-3 2841893

[B25] Murray PR Baron EJ Jorgensen JH Pfaller MA Yolken RH Manual of Clinical Microbiology 2003 8 ASM Press, Washington DC

[B26] Deering RP Orange JS Development of a clinical assay to evaluate toll-like receptor function Clin Vaccine Immunol 2006 13 68 76 10.1128/CVI.13.1.68-76.2006 16426002 PMC1356616

[B27] Hirschfeld M Ma Y Weis JH Vogel SN Weis JJ Cutting edge: repurification of lipopolysaccharide eliminates signaling through both human and murine toll-like receptor 2 J Immunol 2000 165 618 622 10878331 10.4049/jimmunol.165.2.618

[B28] Blander JM Medzhitov R Regulation of phagosome maturation by signals from Toll-like receptors Science 2004 304 1014 1018 10.1126/science.1096158 15143282

[B29] Novak F Hekland DK Avenell A Drover JW Su X Glutamine supplementation in serious illness: a systematic review of the evidence Crit Care Med 2002 30 2022 2029 10.1097/00003246-200209000-00011 12352035

[B30] Singer P Berger MM Vanden Berghe G Biolo G Calder P Forbes A Griffiths R Kreyman G Leverve X Pichard C ESPEN guidelines on parenteral nutrition: Intensive Care Clin Nutr 2009 28 387 400 10.1016/j.clnu.2009.04.024 19505748

[B31] Kessel A Toubi E Pavlotzky E Mogilner J Coran AG Lurie M Karry R Sukhotnik I Treatment with glutamine is associated with down-regulation of Toll-like receptor-4 and myeloid differentiation factor 88 expression and decrease in intestinal mucosal injury caused by lipopolysaccharide endotoxaemia in a rat Clin Exp Immunol 2008 151 341 347 10.1111/j.1365-2249.2007.03571.x 18070149 PMC2276937

[B32] Medzhitov R Janeway C Innate immune recognition: mechanisms and pathways Immunol Rev 2000 173 89 97 10.1034/j.1600-065X.2000.917309.x 10719670

[B33] Qureshi ST Medzhitov R Toll-like receptors and their role in experimental models of microbial infection Genes Immun 2003 4 87 94 10.1038/sj.gene.6363937 12618855

[B34] Akira S Toll-like receptor signalling J Biol Chem 2003 278 38105 108 10.1074/jbc.R300028200 12893815

[B35] Brandl K Gluck T Huber C Salzberger B Falk W Hartmann P TLR4 surface display is increased in septic patients Eur J Med Res 2005 10 319 324 16131472

[B36] Adib-Conquy M Adrie C Fittings C Gasttolliat O Beyaert R Cavaillon JM Up-regulation of MyD88s and SIGIRR, molecules inhibiting Toll-like receptor signaling, in monocytes from septic patients Crit Care Med 2006 34 2377 2385 10.1097/01.CCM.0000233875.93866.88 16850005

[B37] Bihl F Salez L Beaubier M Torres D Larivière L Laroche L Benedetto A Martel D Lapointe JM Ryffel B Malo D Overexpression of Toll-like receptor 4 amplifies the host response to lipopolysaccharide and provides a survival advantage in transgenic mice J Immunol 2003 170 6141 6150 12794144 10.4049/jimmunol.170.12.6141

[B38] Kalis C Kanzler B Lembo A Poltorak A Galanos C Freudenberg MA Toll-like receptor 4 expression levels determine the degree of LPS-susceptibility in mice Eur J Immunol 2003 33 798 805 10.1002/eji.200323431 12616500

[B39] Fabian TC Croce MA Fabian MJ Trenthem LL Yockey JM Boscarino R Proctor KG Reduced tumor necrosis factor production in endotoxin-spiked whole blood after trauma: experimental results and clinical correlation Surgery 1995 118 63 72 10.1016/S0039-6060(05)80011-X 7604381

[B40] Majetschak M Flach R Heukamp T Jennissen V Obertacke U Neudeck F Schmit-Neuerburg KP Schade FU Regulation of whole blood tumor necrosis factor production upon endotoxin stimulation after severe blunt trauma J Trauma 1997 43 880 887 10.1097/00005373-199712000-00002 9420099

[B41] Ogle CK Ogle JD Mao JX Simon J Noel JG Li BG Alexander JW Effect of glutamine on phagocytosis and bacterial killing by normal and pediatric burn patient neutrophils JPEN J Parenter Enteral Nutr 1994 18 128 133 10.1177/0148607194018002128 8201747

